# Low risk for locally acquired Chagas disease in California: A review of human cases and triatomine submissions, 2013–2023

**DOI:** 10.1371/journal.pntd.0013036

**Published:** 2025-04-21

**Authors:** Andrea J. Lund, Marco E. Metzger, Vicki L. Kramer, Anne M. Kjemtrup

**Affiliations:** 1 Infectious Diseases Branch, California Department of Public Health, Sacramento, California, United States of America; 2 Infectious Diseases Branch, California Department of Public Health, Ontario, California, United States of America; IP Montevideo: Institut Pasteur Montevideo, URUGUAY

## Abstract

Chagas disease is caused by infection with the protozoan parasite *Trypanosoma cruzi*, which is carried in the guts of triatomine insects. Transmission typically occurs when infective trypomastigotes in triatomine feces encounter mucous membranes or bite wounds, though it is also possible by food-borne, transplant- and transfusion-mediated, and congenital routes. Most transmission occurs in rural and peri-urban parts of continental Latin America where triatomines often inhabit human dwellings. Triatomines infected with *T. cruzi* are also present across the southern United States, yet relatively few locally acquired infections have been documented. Rather, most reported cases have plausible exposure in Latin America. In California, the widespread distribution of *T. cruzi*-infected triatomines suggests a potential risk of local transmission. Here, we summarize triatomine submissions and human case reports made to the California Department of Public Health between 2013 and 2023. Of 226 triatomines tested, 63 (28%) were positive for *T. cruzi* via PCR; none were linked to any of the 40 human *T. cruzi* cases reported in the same period. Human cases were assessed for likelihood of local transmission. Country of birth, travel history, and location of primary residence suggested non-local transmission for 31 (78%) cases. Local transmission could not be ruled out for the remaining nine (22%) cases. Information on country of birth and travel history were missing from these case reports and prevented full assessment of local transmission criteria, though most of these patients resided within 400 meters of potential triatomine habitat. Despite the presence of triatomines, *T. cruzi*, and human cases in California, statewide data indicates the risk for locally acquired Chagas disease is low.

## Summary

The risk of Chagas disease has historically been limited to continental Latin America, though the presence of the parasite *Trypanosoma cruzi* in triatomine vectors across the southern United States has prompted concerns about the risk of local transmission. In California, records of triatomines infected with *T. cruzi* date to the early 20^th^ century, but few locally acquired cases have been documented statewide. To better understand the burden of Chagas disease in California and assess the risk of local transmission, we summarize eleven years (2013–2023) of triatomine submission and human case data reported to the California Department of Public Health. Almost one-third of triatomines tested positive for *T. cruzi,* though none were linked to human cases. Most of the 40 human cases had plausible exposure in Latin America. The human cases for which local transmission could not be ruled out were missing the demographic information needed to assess local transmission criteria. Despite the presence of triatomines, *T. cruzi*, and human cases in California, the risk for local transmission is low. More complete reporting of demographic information of cases would improve our understanding of local transmission risk and disease burden.

## Introduction

American trypanosomiasis, better known as Chagas disease, is a vector-borne infection caused by the parasitic protozoan *Trypanosoma cruzi*. The *T. cruzi* parasite and its vectors can be found across much of the western hemisphere, from the southern United States to the Patagonian province of Chubut, Argentina [1,2]. Parasite transmission is maintained in nature within a complex cycle involving mammalian reservoir hosts and obligate blood-feeding insects belonging to the subfamily Triatominae (Hemiptera: Reduviidae) [3]. Non-mammalian vertebrates including lizards [4,5] and birds [5] may also be involved in persistence and transmission of *T. cruzi*, but their significance is not well documented. An apparently successful generalist, *T. cruzi* is capable of infecting almost all cell types of most mammalian orders [6], and uses nearly all known species of triatomines for dispersal [7,8].

*Trypanosoma cruzi* trypomastigotes enter triatomines when they take a blood meal from an infected mammal, resulting in permanent infections in the insect’s hindgut. Infective metacyclic trypomastigote stages of the parasite are subsequently shed in the insect’s feces and urine. Transmission to mammals is passive and can occur in a variety of ways. The archetypal route of infection in humans occurs when trypomastigotes excreted on or near the host enter through mucous membranes or open wounds, particularly by scratching feces into the site of a bite [9].

Mammalian infections can also occur by ingestion of whole infected triatomines or their excrement. Predation of infected triatomines or other infected mammals has been proposed as the most efficient means of transmission and is likely responsible for maintaining transmission cycles in wild mammals [4,10–12]. Trypomastigotes can remain infectious inside the abdomens of dead triatomines for days to weeks under certain environmental conditions [13,14]. However, when excreted on surfaces, infectivity is compromised by modest increases in surface temperature [15] and desiccation [16], though their viability and infectivity can be prolonged when deposited in food or drink. Laboratory experiments have documented trypomastigote survival of at least 24 hours in sugar water and various fruit drinks, and as long as 16 days in the refrigerated juice of soursop [17,18]. Oral transmission to humans is an emerging concern in parts of Latin America where vector control initiatives have reduced traditional routes of infection [19–21]. Humans are also at risk for congenital transmission and infection via blood transfusion and organ transplantation from infected donors [8,22,23].

Once established in a mammalian host, the stability and duration of parasitemia varies by infection route, host nutritional status, parasite genotype, and concomitant infections [6,24]. In humans, parasitemia is detectable during the acute phase of infection [25]. Untreated infections become life-long and can cause irreversible, debilitating, and life-threatening damage to the cardiovascular, digestive, and nervous systems [8]. After years of exclusive availability under investigational treatment protocols via the US Centers for Disease Control and Prevention (CDC), two anti-trypanosomal drugs, benznidazole and nifurtimox, are now commercially available in the US to treat both asymptomatic *T. cruzi* infection and symptomatic Chagas disease [26,27]. The effectiveness of treatment is highest in young patients and those in acute and indeterminant stages of infection, with uncertain benefits for older patients with advanced Chagas disease resulting from chronic *T. cruzi* infection [28].

Most human infections with *T. cruzi* are acquired in rural and peri-urban areas of Mexico, Central America, and South America where triatomine species inhabit crevices of walls and roofs of poorly constructed houses. Estimates based on 2010 data suggested approximately 6 million people were infected across 21 Latin American countries, with 70 million people (13% of the population) at risk. The annual number of new cases due to vector-borne and congenital transmission were estimated at 30,000 and 8,700, respectively. This represents a marked reduction – even interruption – of transmission compared with earlier published data. Declining transmission in Latin America has been attributed to vector control initiatives, improvements in home construction and hygiene, blood donor screening, and education campaigns [8].

In the US, Chagas disease is not a nationally notifiable condition, but is reportable in some states including Arizona, Arkansas, Louisiana, Mississippi, Tennessee, Texas, Utah, and Washington [29–31]. While Chagas disease is not required to be reported in the state of California, Los Angeles and San Diego counties recently required county health providers, laboratories, and blood banks to report positive laboratory tests [32,33]. An estimated 300,000 people are living with *T. cruzi* infection in the US, primarily immigrants from Latin America [34]. Eighty locally acquired infections have been reported nationwide since 1955 [35–49], dozens of which were detected since 2007 via risk-based screening in blood and organ donors [22,39]. Investigations of exposure history in positive blood donors have often identified risk factors for infection but precise modes and locations of transmission have been difficult to determine retrospectively [29]. Exposure to infected triatomines was often presumed in these cases, though alternative transmission routes could not be ruled out [35].

Eleven triatomine species are distributed across 27 states in the southern US [50]. *Trypanosoma cruzi* was first described in the US from the gut contents of *Triatoma protracta* collected from wood rat nests in San Diego County, California in 1916 [51]. Since then, natural infections with *T. cruzi* have been documented in nine triatomine species in the US [52]. At least 24 mammalian species are recognized as wildlife hosts of *T. cruzi,* though all mammals are considered susceptible to infection [53]. The prevalence of *T. cruzi* infection in both mammals and vectors varies greatly, likely influenced by local host-parasite interactions. The primary reservoirs of infection vary geographically, with wood rats the most common reservoir in western states, while raccoons, opossums, and armadillos predominate in the eastern US [53].

In California, both parasite and vector have been found over large areas of the state within a wide range of rocky, shrub, and woodland habitats from sea level to over 1,300 meters. Three species of triatomines are native to California: *T. protracta*, *T. rubida*, and *Paratriatoma hirsuta*. Their distribution reflects their reliance on rodents as hosts, commonly infesting the nests of wood rats in the genus *Neotoma* [4,54–58]. *Triatoma protracta* is the most common and widespread species in California, as well as the only triatomine species in which *T. cruzi* infection has been documented in the state (*T. rubida* has been confirmed as a vector elsewhere in the southwest US and *P. hirsuta* infection has been demonstrated under laboratory conditions) [53,56,59]. While these triatomines are generally tied to sylvatic habitats, all three species opportunistically enter homes within their flight range. Despite a decades-long history of reports of triatomine nuisance and bites in California [60–62], only three locally acquired human cases of Chagas disease have been documented: one with confirmed local exposure [45,46] and two others with presumed local exposure [38,49]. In these cases, as with others in the western US [36,48,54], exposure appears to have occurred in or around homes located near triatomine habitat, though outdoor work and recreation (such as camping and hunting) have also been identified as risk factors for local transmission [39,40,44]. The presence of both the vector and causative agent in California, however, suggests a potential risk of locally acquired infections.

Despite not being reportable statewide, the California Department of Public Health (CDPH) occasionally receives reports of human *T. cruzi* infections as well as inquiries from concerned residents regarding triatomines found in and around their homes. Triatomine specimens and human case reports of Chagas disease submitted to CDPH between 2013 and 2023 are presented. We summarize the distribution and infection prevalence of triatomines, classify human case reports under a new surveillance case definition, and assess the potential for local transmission among reported cases. The geographic and epidemiological characteristics of triatomine submissions and human case reports help define the scope of human-triatomine interactions in California and identify the circumstances under which local transmission of *T. cruzi* is most likely to occur.

## Materials and methods

### Triatomine submissions

The CDPH Vector-Borne Disease Section (VBDS) is occasionally contacted by California residents and medical providers who have direct or circumstantial evidence of triatomine bite(s), have collected triatomine specimen(s), and request testing. Starting in 2013, CDPH-VBDS began accepting specimens for identification and testing when circumstances indicated observed or probable indoor exposure (e.g., observed or suspected bite; triatomine found in bed). Submitters provided collection data including address, date of collection, location within the structure where the triatomine(s) was found, and empirical observations including physical reactions to the alleged bite. Eligible triatomines were identified to species and life stage by CDPH-VBDS and sent to the US Centers for Disease Control and Prevention (CDC) for molecular detection of *T. cruzi* infection, and if positive, molecular detection of a human blood meal [63]. Submitters were advised that test results should not be used as the basis for medical decision-making.

### Human case data submission

Human case data were collected as part of public health surveillance activities and as such are not considered research as specified under Section 46.102 of the federal Common Rule (CFR title 45, part 46). The project was deemed exempt by the Committee for the Protection of Human Subjects at the State of California Health and Human Services Agency (Protocol ID: 2025–037). The California Reportable Disease Information Exchange (CalREDIE) is a secure system used by CDPH for electronic disease reporting and surveillance. Typically, local health jurisdictions (LHJs) enter reportable disease information into CalREDIE under disease-specific forms. Because Chagas is not required to be reported in California, LHJs may enter Chagas disease information in CalREDIE using a general form for “Unusual/Other Disease”. Reports of Unusual/Other Disease referencing Chagas disease, *T. cruzi,* and/or American trypanosomiasis submitted between 2013 and 2023 were downloaded from CalREDIE. Clinical, laboratory and epidemiologic information from these records were used to summarize patient demographics as well as evaluate the evidence of infection and how each patient was identified. An assessment of whether infections could have been acquired in California was conducted for patients who were classified as cases.

Patients were identified by LHJs in a variety of ways. Notification following positive blood donor screening came primarily from physicians, whose patients had received letters from the blood banks and prompted them to seek care, though a few patients and blood banks contacted LHJs directly. LHJs were also informed by physicians or laboratories following testing for patients: (1) exhibiting symptoms suggestive of Chagas disease, (2) requesting testing following plausible exposures (e.g., finding a triatomine in the home, or history of residence in a rural area of Latin America), or (3) requesting screening due to other epidemiological risk factor(s), such as positive test results among family members. Other screening for *T. cruzi* infection was performed for patients considering organ or tissue donation, as well as patients who received organ or tissue from a donor infected with *T. cruzi*.

### Data analysis

Evidence of infection was evaluated based on the laboratory results and case investigation notes entered in CalREDIE records. Laboratory results included (a) blood donor screening, (b) serological assays performed at commercial laboratories, and/or (c) confirmatory testing at CDC. In June 2024, the Council of State and Territorial Epidemiologists (CSTE) adopted a surveillance case definition for acute and chronic *T. cruzi* infection and Chagas disease [64]. Available laboratory evidence for each patient was used to classify case-patients according to the CSTE case definition as follows: patients with positive results from multiple serological tests using different antigen preparations, including confirmatory testing at CDC, were classified as confirmed cases; patients with a combination of positive lab results from both blood donor screening and commercial serology were classified as probable cases; and patients with a single positive test result (either blood donor screening or commercial serology) were classified as suspect cases. Patients without positive test results or with negative results of confirmatory testing at CDC were classified as not cases. No clinical criteria were required for case classification.

Assessment of local transmission adapted and modified the criteria used by Lynn et al. (2020) [39] in their review of contemporary local transmission of *T. cruzi* in the United States. For a case to be considered locally acquired, the case-patient had to: (1) have not been born in continental Latin America, (2) have no history of travel to continental Latin America, (3) have no history of tissue or organ transplantation, (4) have no evidence of congenital transmission, and (5) reside within a plausible flight range (400 meters) of suitable triatomine habitat [65]. All five criteria were assessed based on information entered in CalREDIE. If notes indicated maternal birth in continental Latin America, then this suggested evidence of congenital transmission, per criterion (3). The habitat assessment in criterion (5) was the main deviation from Lynn et al.’s (2020) [39] criteria and was performed by first geocoding patient addresses using the *tidygeocoder* package in R (version 1.0.5) [66]. Then, geographic coordinates were plotted in Google Earth Pro (version 7.3.6.9796) and a 400-meter buffer was drawn around each set of coordinates to visually assess for the presence of suitable triatomine habitat. The designation of suitable habitat was given to lands within the 400-meter buffer zone that were partially or wholly unaltered, with native vegetation, and within the known range of triatomines. In summary, exposure outside the US was considered more likely than local transmission for patients born in continental Latin America, with travel history in continental Latin America areas, and/or with a mother who was born or had lived in continental Latin America.

## Results

### Spatiotemporal distribution of entomologic and human data

**Fig 1:**
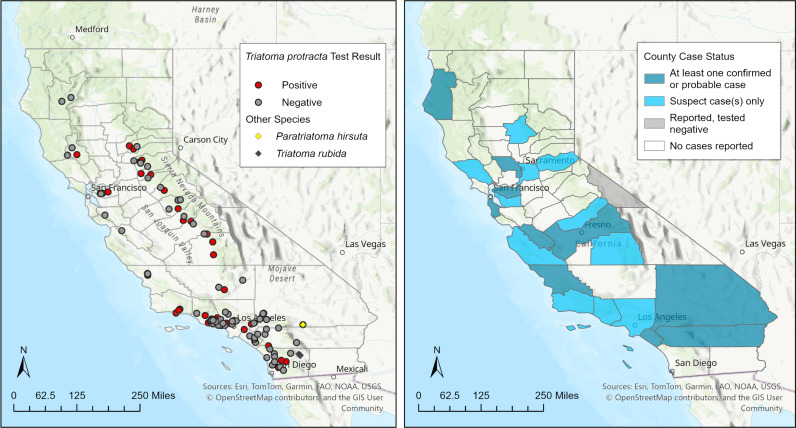
Geographic distribution of triatomine specimens and human case reports submitted to CDPH-VBDS, 2013 - 2023. (Left) Triatomine submissions include 226 specimens with *T. cruzi* infection status indicated in red for 190 adult *T. protracta*. (Right) Human case reports include 52 reports of *T. cruzi* infection and/or Chagas disease investigated in CalREDIE, of which 40 were classified as cases 2013 – 2023. Basemap sources: Esri, TomTom, Garmin, FAO, NOAA, USGS, OpenStreetMap contributors, CNES/Airbus DS, InterMap, NASA/META, NAS/NGS and the GIS User Community [67].

Between 2013 and 2023, CDPH-VBDS received 226 triatomine specimens that met the criteria for testing. Triatomine submissions nearly always were collected from rural properties, or suburban properties adjacent to undeveloped lands with native vegetation. Specimens originated from 25 counties, and were identified as *T. protracta* (190 adults, 22 nymphs), *T. rubida* (1 adult, 4 nymphs), and *P. hirsuta* (8 adults, 1 nymph) (Fig 1). *Triatoma protracta* were submitted from 117 residential homes in all 25 counties at elevations ranging from 37 to 1,350 meters, *T. rubida* from an elementary school office (1 adult) and a residential home (4 nymphs) in Borrego Springs in the low desert region of San Diego County (elevation: 158–186 meters), and *P. hirsuta* from a single residence in the high desert region of San Bernardino County adjacent to Joshua Tree National Park (elevation: 1,060 meters).

In the same period, 52 patients were investigated as potential cases of *T. cruzi* infection or Chagas disease under the “Unusual/Other Disease” category in CalREDIE (Table 1). A median of 4 incidents were reported per year (range: 2–11 incidents per year). Reports came from 22 counties (Fig 1). No human case reports were linked to triatomine specimens submitted to CDPH-VBDS.

**Table 1:** Demographic characteristics of 52 human case reports evaluated for *T. cruzi* infection and/or Chagas disease in California, 2013-2023.

		Method resulting in case identification				
**Demographic characteristic**	**All human case reports**	**Blood donor screening**	**Care seeking**	**Risk-based screening** ^a^	**Other screening ** ^b^	**Unknown**
Reports	52	23	14	7	3	5
Hispanic						
Yes	37 (71%)	19 (83%)	11 (79%)	4 (57%)	2 (67%)	1 (20%)
No	3 (5.8%)	0 (0%)	2 (14%)	0 (0%)	0 (0%)	1 (20%)
Unknown	12 (23%)	4 (17%)	1 (7.1%)	3 (43%)	1 (33%)	3 (60%)
Sex						
Female	26 (50%)	11 (48%)	8 (57%)	3 (43%)	2 (67%)	2 (40%)
Male	26 (50%)	12 (52%)	6 (43%)	4 (57%)	1 (33%)	3 (60%)
Age						
Less than 1 year	1 (1.9%)	0 (0%)	0 (0%)	1 (14%)	0 (0%)	0 (0%)
1 - 20 years	8 (15%)	6 (26%)	2 (14%)	0 (0%)	0 (0%)	0 (0%)
20 - 60 years	33 (63%)	15 (65%)	10 (71%)	3 (43%)	3 (100%)	2 (40%)
60 + years	9 (17%)	1 (4.3%)	2 (14%)	3 (43%)	0 (0%)	3 (60%)
Unknown	1 (1.9%)	1 (4.3%)	0 (0%)	0 (0%)	0 (0%)	0 (0%)
Born in Latin America						
Yes	16 (31%)	9 (39%)	4 (29%)	2 (29%)	1 (33%)	0 (0%)
No	10 (19%)	3 (13%)	2 (14%)	3 (43%)	1 (33%)	1 (20%)
Unknown	26 (50%)	11 (48%)	8 (57%)	2 (29%)	1 (33%)	4 (80%)
Travel to Latin America						
Yes	16 (31%)	9 (39%)	3 (21%)	2 (29%)	2 (67%)	0 (0%)
No	13 (25%)	3 (13%)	5 (36%)	4 (57%)	0 (0%)	1 (20%)
Unknown	23 (44%)	11 (48%)	6 (43%)	1 (14%)	1 (33%)	4 (80%)

^a^Includes asymptomatic patients who sought screening because of epidemiologic risk factor(s), including positive test results among family members, finding a triatomine in the home, or history of residence in rural area of continental Latin America;

^b^Includes patients prospectively screened for organ or tissue donation as well as patients who received organ or tissue from an infected donor.

**Fig 2:**
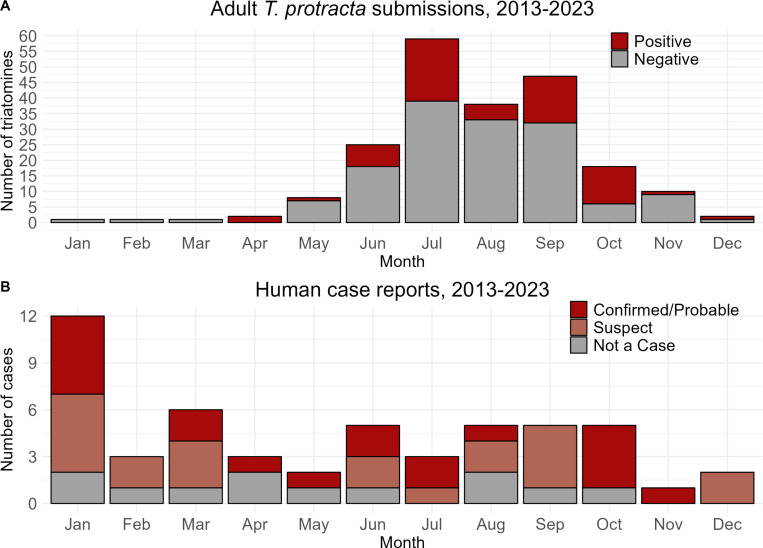
Seasonality of triatomine submissions and human case reports, 2013 – 2023. (A) Seasonality and *T. cruzi* detection in adult *T. protracta* submissions (n = 190; 62 positive, 128 negative) peaked in the summer months. (B) Temporal distribution of human case reports of *T. cruzi* infection and/or Chagas disease investigated in CalREDIE (n = 52; 19 confirmed/probable, 21 suspect and 12 negative/not a case) fluctuated by month without a clear seasonal pattern.

*Triatoma protracta* submissions varied seasonally, with the greatest numbers collected June through October. *Triatoma rubida* and *P. hirsuta* were submitted during the same peak months. Nymphs of all three species were submitted between May and November, and comparatively few specimens were submitted between November and May (Fig 2). Molecular testing detected *T. cruzi* DNA in 62 of 190 adult *Triatoma protracta* (33%); all other specimens (*T. protracta* nymphs, *T. rubida*, and *P. hirsuta*) tested negative. The geographic distribution of infected *T. protracta* was effectively ubiquitous among submitted specimens indicating that the distribution of *T. cruzi*, and hence the potential risk to humans, parallels that of this species in California. Of the 62 *T. cruzi* positive specimens, 31 subsequently tested positive (50%) for a human blood meal confirming physical human-insect interactions (Figs 1 and 2). Human case reports were most common in January (12 reports) and did not exhibit clear seasonality (Fig 2).

### Characteristics of human case reports

Of the 52 patients investigated in CalREDIE between 2013 and 2023, 23 (44%) were identified by blood donor screening (Table 1). Fourteen (27%) were tested upon seeking care for symptoms compatible with Chagas disease while 7 (14%) asymptomatic patients were tested by their providers based on the presence of risk factors (e.g., positive test in a family member or other plausible exposure history). Of the remaining eight patients, three (6%) were identified through other screening (e.g., organ/tissue donors and recipients) and the rest (10%) were identified through unknown means.

Most patients were adults (80% above age 20) and reported Hispanic or Latino ethnicity (71%). Half of the patients were female. Information on country of birth and international travel history was often missing or unknown (Table 1). Country of birth was unknown for 26 (50%) patients, while travel history was unknown for 23 (44%) patients. When data on country of birth and/or travel history were available, patients often had plausible exposure in continental Latin America. Twenty-four (46%) patients were either born in or had travel history to continental Latin America.

### Evidence of infection in human case reports

Nearly all laboratory testing involved serological assays for chronic Chagas disease. A single patient tested negative for acute infection by PCR. Forty (77%) patients were classified as cases (Table 2). Almost one-third (16/40, 31%) were classified as confirmed based on multiple positive test results at CDC. Most patients (21/40, 53%) had a single positive laboratory test and were classified as suspect cases. Of the 23 case-patients identified through blood donor screening, 9 (39%) were classified as suspect with a single positive test result and 9 (39%) were confirmed by CDC. Almost half (9/21, 43%) of patients tested in a healthcare setting (either seeking care for symptoms or asymptomatic patients seeking risk-based screening) tested negative.

**Table 2:** Case classification of 52 human case reports evaluated for *T. cruzi* infection and/or Chagas disease in California, 2013-2023.

		Method resulting in case identification				
**Case Classification**	**All human case reports**	**Blood donor screening**	**Care seeking**	**Risk-based screening ** ^a^	**Other screening ** ^b^	**Unknown**
Reports	52	23	14	7	3	5
Cases	40 (77%)	21 (91%)	8 (57%)	4 (57%)	2 (67%)	5 (100%)
Confirmed	16 (31%)	9 (39%)	4 (29%)	2 (29%)	0 (0%)	1 (20%)
Probable	3 (5.8%)	3 (13%)	0 (0%)	0 (0%)	0 (0%)	0 (0%)
Suspect	21 (40%)	9 (39%)	4 (29%)	2 (29%)	2 (67%)	4 (80%)
Not a Case	12 (23%)	2 (8.7%)	6 (43%)	3 (43%)	1 (33%)	0 (0%)

^a^Includes asymptomatic patients who sought screening because of epidemiologic risk factor(s), including positive test results among family members, finding a triatomine in the home, or history of residence in rural area of continental Latin America;

^b^Includes patients prospectively screened for organ or tissue donation as well as patients who received organ or tissue from an infected donor.

### Local transmission assessment

**Fig 3:**
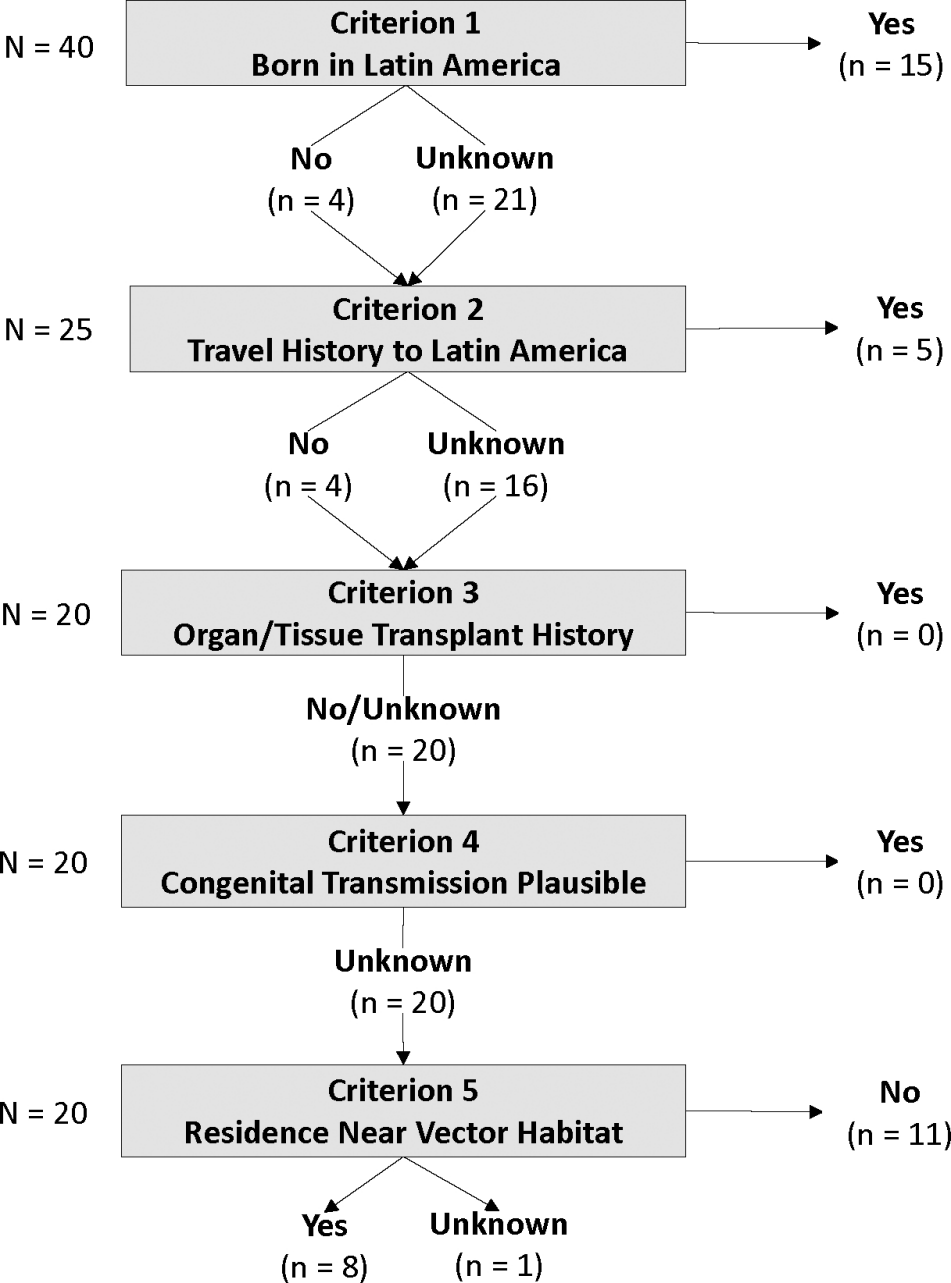
Local transmission assessment of Chagas cases reported in California, 2013 – 2023. Five criteria adapted from Lynn et al. [39] were applied to the 40 human case reports that were classified as cases according to the surveillance case definition. Cases classified as confirmed, probable, and suspect were included in the local transmission assessment.

The five criteria for discerning local transmission were assessed with information available in CalREDIE (Fig 3). Information on country of birth and travel history ruled local transmission as unlikely for 20 (50%) case-patients. Congenital transmission could not be assessed, as maternal country of birth was not entered or noted for any case-patients, and no notes indicated organ or transplant history for any of the case-patients whose country of birth or travel history suggested non-local transmission. Of the remaining 20 case-patients, local transmission was deemed unlikely for 11 (55%) because their residence was located more than 400 meters from suitable triatomine habitat, precluding local exposure risk. In all, local transmission could not be ruled in or out for nine (23%) case-patients. However, CalREDIE records were missing information on all criteria except for residence near vector habitat for all but one (8/9, 89%) of these case-patients. While local transmission could not be ruled in or out for the eight case -patients residing near vector habitat, the likelihood of local transmission could not be thoroughly assessed due to missing information on local transmission criteria.

## Discussion

Eleven years of triatomine submissions and human case reports demonstrate that local transmission of *T. cruzi* is possible but rare in California with a low risk to the public. One-third of triatomines submitted to CDPH-VBDS were infected with *T. cruzi,* and half of those had evidence of a human blood meal, though none were linked to human case reports. Most human case reports involved the detection of chronic *T. cruzi* infection in patients who were born or had travel history in continental Latin America. Local transmission was considered unlikely for most case-patients with no or unknown travel history because they lived in urban or suburban areas beyond plausible flight distance from suitable triatomine habitat. Most of the case-patients for whom local transmission could not be ruled out were missing information on all local transmission criteria except for residence near vector habitat. We were also unable to evaluate potential local exposures to triatomines that may have occurred outside the home through work or recreational activities.

Detection of *T. cruzi* infection in adult *T. protracta* specimens submitted to CDPH-VBDS was comparable to previous studies of this species in California dating back to the early 20^th^ century [51,68,69]. These detections are consistent with active cycles of transmission between *T. protracta* and wildlife reservoirs, particularly rodents from the genus *Neotoma*. Such transmission among wildlife is likely maintained by ingestion of infected triatomines or predation on other infected mammals, a mode of transmission that is far more efficient than the route requiring vector feces to cross skin or mucosal barriers [10–12]. Intense and efficient transmission between *T. protracta* and their wildlife hosts may allow *T. cruzi* to circulate in California without posing substantial risk to people, whose exposure depends on proximity to triatomine habitat and the less efficient fecal transmission route.

Successful transmission of *T. cruzi* from triatomines to humans via vector feces requires a unique combination of events. First, an infected vector must defecate sufficiently close to a bite site or mucous membrane whilst or shortly after feeding. Then, live trypomastigotes in the feces must be moved by the host to the susceptible entry point (e.g., via scratching), and finally, the trypomastigotes must successfully pass into the body to initiate infection. This route is estimated to require between 900 and 4,000 encounters with triatomines for human infection to occur, making it inefficient even in areas of Latin America with high levels of transmission by triatomine species that inhabit human homes [70,71]. Few people in California experience this level of exposure, and concern for acute, locally acquired infection should focus on residents with numerous and continued encounters with triatomines.

There is little evidence that the triatomines native to California will occupy human homes in the same way that important vectors in Latin America do. The most abundant and widespread species in California, *T. protracta*, is sylvatic and can persist within relatively small areas of undeveloped land that supports native host animals and vegetation, including open space within the vast urban and suburban matrix of the Greater Los Angeles metropolitan area. In contrast, *T. rubida* and *P. hirsuta* are only found in southeastern desert regions of the state where the human population is far less dense. Rural and suburban homes near triatomine habitats are where humans are most likely to encounter triatomines. On warm evenings just after dark, adult *T. protracta* and *T. rubida* (and likely *P. hirsuta*) fly from their habitats towards artificial lights, presumably in search of hosts. These flights result in seasonal and opportunistic entry into homes, most commonly through and under doors [54–57,61,65,72,73].

Triatomine submissions to CDPH-VBDS were consistent with these patterns of seasonal and opportunistic entry into homes. The timing of submissions matched that of typical temperature-dependent adult flights. Many submitted specimens of *T. protracta* were found dead inside homes despite the presence of potential hosts. This could be a result of adults arriving at lights near death from starvation [72,73] and also may indicate that *T. protracta* is not particularly well-suited to life indoors. Either conclusion is supported by the small number of nymph submissions. Even with incidental invasion of human homes, the detection of human bloodmeals in half of infected specimens indicates that triatomines in California feed on humans when available. Thus, the risk of local exposure to *T. cruzi* is present in California wherever *T. protracta* is found, but especially for people whose homes are located near suitable triatomine habitat, who frequently find *T. protracta* in their homes, or who spend considerable time outdoors in triatomine habitat.

Human case report data also agree with previous reports of locally acquired human cases: the risk is very low for the majority of Californians [38,45,46,49]. Various factors may explain this, including the prevalence and likelihood of human-triatomine encounters, the behavior of triatomines, and the survivorship of excreted trypomastigotes. *Triatoma protracta’*s preference for animal hosts limits their encounters with humans. A systematic review of triatomine blood meal analyses, which found that *T. protracta* fed primarily on wood rats and infrequently on people, supports this [50]. Homes near triatomine habitat and their preferred hosts are most often located in rural areas, which accommodate a small and shrinking proportion of California’s human population [74]. Human-triatomine encounters are further limited by housing not typically conducive to triatomine intrusion. Solid walls and roofs, with doors and windows sealed for air conditioning, make entry and colonization difficult [75].

When human-triatomine contact does occur, the defecation behavior of *T. protracta* (and to a slightly lesser extent *T. rubida*) is likely to limit contact with infective feces. These species delay a large proportion of defecations until after they have finished feeding and moved away from the host [76–78], though infection with *T. cruzi* may shorten this interval [79,80]. In contrast, important vectors in Latin America tend to defecate sooner and more frequently after feeding, placing potentially infective trypomastigotes on or near the host and the bite wound [81,82]. Furthermore, transmission via contaminated surfaces is limited by the fragility of the trypomastigotes excreted in triatomine feces. These organisms require moisture to survive and quickly lose motility and infectivity as triatomine excrement dries [16]. Low relative humidity and surface temperatures only moderately above ambient will rapidly compromise their infectivity [15], as will soap and common disinfectants [83]. With evidence supporting a very low risk of transmission of *T. cruzi* to humans in California, triatomine bite allergy poses a greater medical risk, as reactions to salivary compounds injected during feeding have been documented for all three triatomine species present in the state [57,65,84,85].

Across the United States, the introduction of blood donor screening in 2007 allowed for the detection of *T. cruzi* infections with local transmission. Between 1955 and the mid-2000s, just seven cases of locally acquired Chagas disease had been documented nationwide [53]. Since 2007, blood donor and vector exposure follow-up studies identified dozens of additional cases, with suggested local transmission in eight states [39,40,86]. Most case reports in jurisdictions where Chagas disease is reportable arose from blood donor screening [29], a pattern we also found in California. However, clear evidence of contact with triatomines or with trypomastigote-contaminated feces was often absent. The limited exposure assessment of these retrospective studies left the source and route of infection up to speculation. Since 2008, detections of *T. cruzi* via blood donor screening have declined [87], prompting Massachusetts to remove Chagas from its list of reportable conditions in 2014 [29] and the Association for the Advancement of Blood and Biotherapies to discontinue their Chagas Biovigilance Network in 2020 [88]. In both cases, identification of transfusion-transmitted *T. cruzi* infection was so rare that the public health benefits of surveillance no longer outweighed the costs.

Even so, *T. cruzi* infection and Chagas disease remain a public health concern in California. Infections among California residents that were acquired outside of the country may still represent a substantial burden of disease, especially in jurisdictions with large populations of Latin American descent. In these jurisdictions, public health surveillance may facilitate appropriate diagnostic testing and connect patients to care. Because treatment is most successful when initiated early in the course of illness, even before symptoms develop, public health follow-up on diagnostic testing may promote care-seeking that would otherwise not occur. Accordingly, such follow-up will have the greatest impact for cases of acute Chagas disease, as subsequent treatment is more likely to be successful. Our analysis suggests, however, that public health surveillance is most likely to capture chronic cases, for which treatment may or may not be advised. The uncertain benefits of public health surveillance introduce complexity to the issue of Chagas reportability in California and elsewhere. In addition to follow up being a time-consuming undertaking, public health agencies may have limited capacity to connect patients to care, and their efforts may be further hampered if at-risk populations face other structural barriers to care. As a result, we recommend that public health agencies balance risks and benefits when deciding whether to initiate public health surveillance for *T. cruzi* infection and Chagas disease in their jurisdictions.

In other western states where Chagas disease is reportable, public health surveillance has revealed relatively few cases, which appear to be similarly dominated by chronic infections acquired outside the US. Since initiating Chagas reportability in 2008, the Arizona Department of Health Services has reported just 38 cases of Chagas disease [89], while the Utah Department of Health and Human Services reported 4 cases between 2018 and 2020 [90]. By comparison, the Texas Department of State Health Services has reported 251 cases of Chagas disease in 10 years of surveillance. All but three of these cases were chronic infections and 19% were considered locally acquired [91]. The disparity between Texas and other western states, including California, may be due to the greater number of triatomine species present in Texas as well as more reports of human-triatomine encounters [50,92].

Our analysis of triatomine submissions and human case reports for Chagas disease in California is not without limitations. Because *T. cruzi* infection and Chagas disease are not reportable statewide, human case data have been reported opportunistically by LHJs. As a result, the information available for case classification and assessment of local transmission varied considerably. Case reports were often missing demographic data that limited our assessment of local transmission criteria. Some had incomplete laboratory data entered, and the retrospective nature of our review limited our ability to conduct further follow up with local case investigators and case-patients, including assessment of exposures through outdoor work or recreation. While occupational and recreational exposure is possible, residential exposure is most likely given triatomines’ nocturnal flight and feeding behaviors. Moreover, the predominance of chronic Chagas disease among human case reports made it difficult to discern when, where, and how infection was acquired.

Our analysis also excludes data from Los Angeles and San Diego counties, two metropolitan areas with large at-risk populations [34] but have separate reporting systems from the statewide system [32,33]. Because these jurisdictions recently made Chagas reportable, we were not able to include their data in this analysis. Once a Chagas-specific case report form is available, these jurisdictions will be able to submit case reports of Chagas disease to CDPH. This, combined with opportunistic reporting from the remaining 59 local health jurisdictions, means that our analysis likely underestimates the true number of cases of *T. cruzi* infection and Chagas disease in California during the study period.

Currently, there is not a strong case for making Chagas disease reportable across the entirety of California. The most beneficial public health actions for this condition are to ensure that patients receive appropriate diagnostic testing and are connected to treatment and care, as necessary. Statewide reportability would impose a burden on small, resource-constrained LHJs, with uncertain opportunity for public health action. Because the burden of Chagas disease in California seems to be borne primarily by residents who acquired infections in continental Latin America, case investigations are unlikely to reveal the precise circumstances of exposure, obviating the need for timely environmental investigation or intervention. And while the risk of local transmission is highest for people living in rural areas and suburban peripheries, most cases are likely to surface in metropolitan areas with large at-risk populations.

In the absence of statewide reportability, standardizing data collection and case classification will improve follow-up on human case reports in jurisdictions that have the capacity to conduct case investigations. The former will ensure that the demographic information necessary to assess local transmission risk is routinely collected. Data on country of birth (for both the patient and their gestational parent) and travel history are essential for ruling out local transmission, while data on triatomine encounters and proximity to suitable triatomine habitat can verify the possibility of local exposure, especially for acute cases. Including fields for these data in a standardized form will improve the availability and quality of surveillance data. In turn, the national case definition, approved by CSTE in June 2024, will further improve the consistency of case classification across jurisdictions. Together, these measures will clarify the impact of Chagas disease on California’s population as well as its medical system and lay groundwork for further exploration of statewide reportability in the future.

## Conclusions

In summary, our data reveal no reports of Chagas disease among California residents who submitted triatomine specimens to CDPH-VBDS. These data are consistent with the handful of existing reports of local transmission and suggest a low risk of acquiring *T. cruzi* infection from triatomines in California. Most human case reports involved the detection of chronic infections in people who likely acquired their infections in Latin America. An average of four cases per year were identified statewide, though the opportunistic nature of this reporting likely underestimates the true number of cases. Future efforts to standardize case reporting, including in two jurisdictions that recently made Chagas disease locally reportable, will further the health equity goals of connecting Californians infected with *T. cruzi* to appropriate care and improve our understanding of the burden of disease and risk of local transmission in California.
